# Supplementary material for: Using portraits to quantify the changes of generalized social trust in European history: A replication study
Source: PLoS One. 2023 Sep 15;18(9):e0289741. doi: 10.1371/journal.pone.0289741 (PMC10503726; doi:10.1371/journal.pone.0289741)
Supplement: Attachment — Submitted filename: POSE Rebuttal.docx [file pone.0289741.s001.docx]

Dear Anastassia Zabrodskaja,

Please find a response to the points raised by the two reviewers. We are grateful for their time and their attention.

Best,

Lou Safra, Léonard Guillou and Nicolas Baumard

Reviewer #1: The authors have addressed the substantial comments from the previous review. I think this has greatly improved the manuscript - particularly in terms of attenuating any overly bold claims about the ability of the methodology to detect actual trustworthiness (as a personality trait). However, I do have some outstanding comments, some driven by the revisions to the manuscript and others inspired by a second examination of the paper. Once again, my substantive comments are that there should be more explicit acknowledgement of the conceptual limitations of this approach and of the use of algorithms to model human judgements.

> We would like to thank Reviewer 1 for this comment and are happy that no ambiguity is present in the current version of the manuscript. We would also like to reiterate that our method has always aimed at approximating human evaluations of perceived trustworthiness and not sitter’s actual trustworthiness.

I thank the authors for providing a view-only link to the OSF project. I notice however that there are some files referenced in the scripts that are not available on the project, making it impossible to recreate the power analysis reported. I also notice that the scripts as available do not directly replicate the analysis reported in the manuscript - the model coefficients in Table 2, for example, are as reported for dominance, gender, and age, but with different coefficients for the intercept and effect of date. I suspect this may have to do with the default format of the predictor across different versions of R but I mention it so that the authors can double check

> We would like to thank Reviewer 1 for having checked on our scripts so carefully. The statistics reported in the manuscript indeed taken year as a scaled variable, we included the corresponding line in the online script. We also included links to the relevant datafiles for the power analysis. We re-ran all our analyses using the latest version of R which led to minor changes in the coefficient values, but not the level of significance, of the mixed effect regressions.

The authors acknowledge at one point that although GDP is a useful measure for tracking long-term changes in across centuries and countries, it is only a partial assessment and as you get earlier in history the data become more sparse and uncertain. I think it is also worth acknowledging that the same could also be true for trustworthiness. While there is increasing evidence that first impressions of faces based on physical features are robust across naturalistic images and across cultures (see <https://doi.org/0.1177/0146167217744194>), it is still a strong assumption--and a central one to the authors' work--that such physiognomy-driven impressions are a human universal and always have been. While I do not think this assumption is unwarranted, it is worth acknowledging and considering alternative hypotheses (e.g. the reason that the prevalence of 'trustworthy' facial features and expressions increases over time is due to increased reliance on physiognomic cues to trust rather than, say, postural or setting cues such as praying).

> We agree with Reviewer 1 that it is a possibility that other types of cues were privileged in the past in addition to facial features to signal cooperative intents, although the non-linear patterns identified in our databases suggest that at least, if it is the case, facial signals have not monotonically replaced other types of signals in the history of portraits. In the revised version of the manuscript, we discuss this possibility in the Discussion section.

**4. General discussion**

Finally, it should be recognized that portraits contain much more information than just the facial features we analyzed in the present study. Pose, costume, the presence of certain objects or animals have been used throughout art history to signal certain statuses, characteristics, and intentions. For example, dogs are known to have been used in official portraiture as symbols of loyalty. Therefore, by relying solely on facial features, our analyses omitted culturally important information contained in portraits. One consequence of this methodological choice is that we cannot rule out the possibility that cooperative intentions were primarily communicated by means other than facial features in a more distant past and that their importance has gradually been replaced by that of facial features in more recent periods, a process that could explain the increase in perceived facial trustworthiness in more recent portraits. However, while the question of the robustness of first impressions of faces across populations and cultures requires further investigation, these other symbols are highly culture-specific (as in the case of parrots; Verdi, 2007), which precludes their use in longitudinal or comparative studies.

**Reference**

Verdi, Richard. *The parrot in art: from Dürer to Elizabeth Butterworth*. Scala Books, 2007.

It would also be worth considering the limitations of automated feature extraction. I understand that the Open Face algorithm is optimised on computer-generated avatars that are specifically designed to vary in perceived trustworthiness and perceived dominance. The use of this algorithm to estimate human judgements of real-face photographs has been validated, but its application to generated faces presents new questions and challenges. Do lower ratings of perceived trustworthiness in earlier portraits reflect a lower motivation to be seen as trustworthy, or do they reflect increased difficulty for the algorithm in extracting and identifying the relevant facial features? Is an increase in perceived trustworthiness over time related to growing democratic norms or is it just a side-effect of a cultural shift towards realism and dynamic expression in portraiture providing more information to the algorithm?

> We would like to thank Reviewer 1 for pointing out that this aspect of our method is unclear. Open Face is an algorithm initially developed by Dr. Tadas Baltrusaitis, trained on naturalistic images. We used OpenFace algorithm on computer-generated avatar faces to develop a random-forest algorithm converting the initial output of OpenFace (facial action units) into scores of modeled perceived facial trustworthiness and dominance. However, it is true that some portraits, notably due to their style or to the posture of the sitter, were not accurately detected by OpenFace. This is why we used a multi-raters manual check of the quality of the detection of the landmarks of each portrait, that was used as a weight in our analyses. Nonetheless, it is possible that some paiting features, such as the level of realism, could artificially decrease the modeled perceived facial trustworthiness scores generated by our algorithm. Nonetheless, if it were the case, one could have expected monotonic changes of these scores with time which is not what we found in neither database. We discuss this important hypothesis in the revised version of the manuscript.

**4. General discussion**

Finally, it is worth noting that the non-monotonic changes in modeled perceived facial trustworthiness evidenced in our databases suggest that the reported evolutions are not due to the mere fact that the facial features present in more recent portraits provide more information that can be processed by our method.

I am not sure how the authors could address this empirically without substantial extra work to validate the performance of the algorithm on non-natural faces, but one approach could be to examine whether algorithmically computed ratings of dominance (or another social judgement) also increase over time or whether this change is specific to perceived trustworthiness. If both ratings increase over time this could indicate that the results are driven by an increase in the amount of information available to the algorithm rather than a specific increase in motivation to appear trustworthy. I see that the authors included dominance as a factor in the models predicting trustworthiness but this is not fully justified beyond the fact that dominance is a known confound. This should be explained further, in any case.

> We would like to thank Reviewer 1 for this suggestion. In addition to the fact that the increase in modeled perceived facial trustworthiness is non-monotonic, which suggest that the reported effect is not only due to information effect, we did not find any significant increase of modeled perceived facial dominance with time in our databases (models with age, gender and time as predictors: Sovereign database: *b* = 0.04 ±0.04, *t*(253) = 1.02, *p* > .250; Versailles database: *b* = 0.06 ± 0.04, *t*(760) = 1.47, *p* = .141), providing further evidence in this direction. However, as perceived facial dominance is likely to carry a conceptually distinct signal compared to perceived facial trustworthiness, we only included this analysis in the peer review history of the paper. Nonetheless we further present the interest of including modeled perceived facial dominance in our models in the revised version of the manuscript.

**2.1.3 Statistical analyses:**

Crucially, as modeled perceived facial dominance was computed in a similar way as modeled perceived facial trustworthiness, including this variable in our model enable to also control for the specificity of the changes in our dependent variable compared to other facial-feature driven trait perceptions modeled using our method.

Some more minor comments:

There are a number of typographical and grammatical mistakes that the authors will want to check because PLOS does not copyedit manuscripts before they go to print and relies on reviewers and authors to correct any typographical errors themselves. Some of these are minor, such as the overuse of the definite article where it is not necessary (e.g. the first 'the' in the following sentence: "The portraits reveal the way the person who ordered the portrait wants to be perceived..."), but some could disrupt reader comprehension. I identify some of these here and there but the authors should check through carefully for more

p.2: In the second paragraph of the general introduction, in the new addition, the voice changes from the second person "You" to first person "We". I usually find it preferable to reserve "We" for talking specifically about decisions and actions made by the authors.

p.2: "You can choose to look young or old, to be natural or to correct facial imperfections" - I understand what the authors mean here, but "imperfections" is perhaps a bit strong and may be perceived as inappropriate or even offensive. I would clarify that such "corrections" involve editing or removing facial features that do not conform to contemporary aesthetic standards

p.4: "Another potential limits of Safra et al... is that the only study on non-English portraits relied on the use of multiple databases and thus potentially varying in their resolutions" - change to "Another potential limit... relied on the use of multiple database and so included portraits that potentially varied in their resolution"

> We would like to thank Reviewer 1 for their careful reading of our manuscript. We corrected all the indicated issues and proceeded to a throughout check of the manuscript.

p.4: "This gives the opportunity to free oneself from any intuition or cultural bias related to clothing, hairstyle, or the background of the portrait" - although it is still subject to other cultural biases determine by the constitution of the data on which it was trained

> We agree with Reviewer 1 that the model is subject to the cultural biases embedded in the training set. In the revised version of the manuscript, we precisely indicate that we are discussing intuitions related to historical cues which can create major biases in our study.

**1. Introduction**

This gives the opportunity to free oneself from any intuition or cultural bias related to historical cues, such as clothing, hairstyle, or the background of the portrait (Todorov et al., 2015).

p.4: "Our candidate is economic resources." - the details and predictions of the modernisation theory could be reiterated here to clarify why economic factors are expected to relate to self-presentation

> We agree that the link with previous work should be highlighted here. However, while the modernization theory makes strong claim on the mechanisms linking economic resources and social values, our studies just aimed at replicating the association between GDP and modeled perceived facial trustworthiness in portraits reported by Safra et al. (2020). In the revised version of the manuscript, we more clearly presented the rationale for this analysis.

**1. Introduction**

Based on the modernization theory, which proposes a link between economic development and increase in cooperation at the society level and which was already successfully on contemporary data (Inglehart, 2018; Inglehart & Welzel, 2005; Lipset, 1959) and in the survey conducted by Safra et al. (2020) we tested the effect of economic resources.

p.5: "The quality checks performed on this sample..." I recommend adding a reference to the later section (e.g. "see Statistical Analysis section") so that readers know this is not something they need to look for in the preregistration documents

> We would like to thank Reviewer 1 for this suggestion, we included the reference in the revised version of the manuscript.

p.5: "In order to have samples similar to the one of sovereigns, portraits dated later than 1950 were excluded before drawing our subsamples." - this is not clear and I do not understand what the authors did here, considering that it appears there were post-1950 portraits in both datasets?

> We agree with Reviewer 1 that this sentence was ambiguous. The post-1950 portraits were in the National Portrait Gallery database analyzed in Safra et al. (2020). We made this point clearer in the revised version of the manuscript.

**2.1 Material and Methods**

In order to have samples similar to the one of sovereigns, portraits dated later than 1950 were excluded from the National Portraits Gallery database before drawing our subsamples.

p.7: While the authors do mention race when describing the second dataset, it would also be helpful to mention that all faces were White in the description of the final sample

> We added this precision in the method section.

**Methods 3.2.2**

Although this element is an important limit of the used method, in the specific context of our studies it has a very limited impact on the robustness of our results given the very smal percentage of non-White faces in our database of historical European portraits. This issue was even absent in the European sovereign database (Study 1) as it contained only White faces.

p.13: "A correlation between GDP per capita and perceived facial trustworthiness was also found" - also in the first paragraph of the discussion, the authors should take care to remind readers that this relationship did not survive when time was also included in the model. Also in the following paragraph, "our results replicate the increase of perceived facial trustworthiness with time and economic affluence found by Safra et al" - but the current results do not replicate that relationship, and the only time such a relationship is observed it appears to be due to a confound

> We agree with Reviewer 1 that this result should be discussed direction in the discussion section of this study, it is now done in the revised version of the manuscript.

**2.3 Discussion**

A correlation between GDP per capita and modeled perceived facial trustworthiness was also found. However, this association did not survive to the inclusion of time as a controlling variable, suggesting that this association was rather due to the general increase of GDP per capita throughout the studied period.

p.17: The description of the methods and results gets quite confusing with so many different things being compared. For example, what was the final sample size subjected to analysis in section 3.3.3?

> We would like to thank Reviewer 1 for pointing out this issue. In the revised version of the manuscript we clearly indicated the databases on which each analyses were conducted.

**2.1.3 Statistical analyses**

However, for 51 portraits, GDP per capita could not be retrieved, analyses including GDP per capita were thus conducted on a subsample of 915 portraits.

**3.2.1 Data collection and pre-processing**

When possible, we also calculated the age of the subject of the portrait and identify her/his gender. Gender was manually coded based on the names of the sitter and could have been retrieved for 1,399 portraits. However, it should be noted that, contrary to the National Portrait Gallery database, because the portraits do not necessarily represent well-known personalities, several birth and death dates were not available online. In the analyzed dataset, the age could be retrieved only for 836 portraits. Age and gender could be retrieved for 764, which constituted the final database on which the analyses were conducted (Table 4). Concerning the economic data, we collected GDP per capita estimates from the Maddison project (Bolt & van Zanden, 2020). A GDP per capita value was retrieved for all the portraits of the final database.

p.17: "the analysis of the distribution of the portraits reveals only very few portraits dated before 1600 or after 1850 (Kolmogorov-Smirnov test..." - the KS-test does not back up the statement it follows, as no reference is made to the normality of the distribution

> We agree with Reviewer 1, we replaced this test by a description of the percentage of paintings produced in each period.

**3.4. Discussion**

Indeed, although our dataset included portraits which were produced between 1483 and 1950, the analysis of the distribution of the portraits revealed only very few portraits dated before 1600 or after 1850 (2 % and 9% respectively),

p.17: "Study 1 did replicate the results..." - Once again, this implies that the current findings support a relationship between perceived trustworthiness and yGDP, which is not the case

> We agree with Reviewer 1 and in the revised this sentence accordingly.

**4. General discussion**

Study 1 did replicate the results on the association between time and modeled perceived facial trustworthiness, using a database controlled for the social status of the sitters. However, Study 2 failed to replicate these results and in both databases, the effect of economic development was not significant when time was included as a covariate.

p.18: "This result [failure to replicate] highlights the limits of this new methodology" - I would advise rewording this as by my personal reading, this suggests that the authors are undermining their own methodology because it does not support the theory whose predictions they set out to test. This gives an impression of 'moving the goalposts' rather than considering what implications this failure to replicate have for the theory itself. I do not think that this is what the authors intended, and so they should clarify

> We agree with Reviewer 1, and as discussed in the manuscript, that this failure to replicate has not only implications for the methods but regarding the initial results. We more clearly state this point in the revised version of the manuscript.

**4. General discussion**

This result highlights the limit of the initial findings and of this new methodology.

p.19: "This can notably contribute to the debate on the causes of democratic revolutions in Europe during the modern period" - I am unfamiliar with this debate or the literature, but this seems like quite a strong claim. It might be helpful to clarify what, exactly, this contribution might look like

> There has been a long debate in the social sciences about the ultimate cause of the democratic revolutions of Western Europe in the modern period, with some emphasizing economic development and others suggesting an important role for institutions. Here, we contribute to this debate by suggesting that the rise of democracy was preceded by a change in the psychology of the people, as evidenced by the way in which they were portrayed during this period.

**4. General discussion**

This can notably contribute to the debate on the causes of democratic revolutions in Europe during the modern period, and in particular the role of economic development as an ultimate factor, and psychological changes as a proximate factor in fostering institutional change (Acemoglu & Robinson, 2012; Baumard, 2018; Fukuyama, 2011).

p.19: "Cognitive studies of social impressions in the arts are thus one more a new tools..." - this is poorly worded and I am not sure what exactly the authors are trying to say

> Thank you for pointing out this issue. We rephrased this sentence in the revised version of the manuscript.

**4. General discussion**

Using insights from cognitive science studies on first impressions from faces to analyze the variations in artworks are thus one more a new tools in the emerging field of quantitative psychological history (Acerbi et al., 2013; Baumard et al., 2022; Martins & Baumard, 2020, 2022; Seligman, 2022).

**Reviewer #2:**

*1. The study presents the results of original research.*

*Yes.

2. Results reported have not been published elsewhere.*

*A Google Scholar search did not turn up any evidence that this work has been previously published.

3. Experiments, statistics, and other analyses are performed to a high technical standard and are described in sufficient detail.*

*- For the random forest model, it's unclear whether the authors are using one of their models from their previous work, or have developed a new model for the current paper.*

> Thank you, in the revised version of the manuscript indicated that we used the random forest model used in this paper was trained using the exact same method and database as the one reported in Safra et al. (2020).

**2.1.2. Computation of perceived trustworthiness and perceived dominance levels**

Level of perceived trustworthiness and perceived dominance of faces were computed based on the facial action units automatically extracted by Open Face 3.1 (<https://github.com/TadasBaltrusaitis/OpenFace>) and transformed into perceived facial trustworthiness and perceived facial dominance evaluation scores using a random forest algorithm optimized on the avatars controlled for perceived trustworthiness and perceived dominance generated by FaceGen (Oosterhof & Todorov, 2008), trained using the exact same method and database as the one reported in Safra et al. (2020).

*- Table 5 shows the gender/age quality check regression results, but the reference in the text (§3.3.3, page 56 of the review packet) suggests it should show the analytic regression results (effects for time and GDP).*

> Thank you, the reference to Table 5 has been removed in the revised version of the manuscript.

*4. Conclusions are presented in an appropriate fashion and are supported by the data.
To my mind, the crucial question for both the current paper and Safra et al. (2020) is whether and to what extent the authors' method measures trustworthiness. Insofar as they are not measuring trustworthiness, their conclusions about trustworthiness cannot be supported.*

*Following their previous work, the authors propose that the "perceived trustworthiness" of figures in historical portraits can be measured using a random forest (RF) model trained on facial action units and the first principal component of an exploratory PCA of descriptions of synthetic faces conducted by Oosteroff and Todorov (2008). This measurement technique relies on several crucial assumptions:*

*1. There is a universal/culturally independent construct of trustworthiness*

*2. Oosteroff and Todorov's (2008) first principal component operationalizes this construct (rather than age, gender, some uninterpretable composite, etc.)*

*3. Accuracy of the RF model at predicting ground truth/human assessments of trustworthiness
4. Extrapolation from the early 21st century WEIRD samples used by Oosteroff and Todorov (2008) and others to contemporaneous viewers of the historical portraits*

*5. That trustworthiness of portrait subjects was communicated primarily by facial features, rather than other features of the subject such as pose and dress*

*6. And rather than other features of the portrait, such as the presence of other characters, inanimate objects, or background and their relationship to the subject*

*Assumption 3 was acknowledged as a weakness in Safra et al. (2020): they reported this accuracy as only r = 0.22. Assumption 4 was also identified as a key assumption ("the assumption that facial cues that are used as cues to assess perceived trustworthiness are shared across time"), with the authors writing that "further work is needed to fully test this assumption." Criticisms related to the other assumptions were raised in the peer review of Safra et al. (2020) (made available by the publisher at* [*https://static-content.springer.com/esm/art%3A10.1038%2Fs41467-020-18566-7/MediaObjects/41467_2020_18566_MOESM3_ESM.pdf*](https://static-content.springer.com/esm/art%3A10.1038%2Fs41467-020-18566-7/MediaObjects/41467_2020_18566_MOESM3_ESM.pdf)*) and public discussion after Safra et al. (2020) was published.*

*In this light, I would have expected follow-up work on Safra et al. (2020) to systematically study (and, hopefully, empirically confirm) these assumptions, conceptually and psychometrically validating the authors' technique. Based on Safra's Google Scholar profile, the authors have not done this work. But until this work has been done, any conclusions about trustworthiness based on the authors' methods are premature. In particular, attempting to replicate the headline finding of Safra et al. (2020) does nothing to support these crucial assumptions.

On the other hand, "the authors should have done a different study" is generally considered an unfair criticism, and the current project was a reasonable replication attempt of the headline finding of Safra et al. (2020). Whatever it is that the authors' technique measures, it was measured the same way as in Safra et al. (2020) and was correlated with time and GPD in Study 1, but not in Study 2.*

*I would be more supportive of publishing the current paper if
(1) the term "perceived trustworthiness" were replaced with a more hedged term, such as "estimated trustworthiness" or "modeled trustworthiness";*

*(2) the authors noted that there are substantial unanswered questions about the validity of their measurement technique;*

> We would like to thank Reviewer 2 for reading our manuscript. We agree that other cues, such as posture, outfit and objects are also well-known for having been used by portraitists for providing information about the sitter’s character and intentions. (ref d’histoire de l’art). Analyzing only the facial features of portraits thus limits the quantity of information that is extracted from the portraits. Further, it is indeed possible that in specific periods, other cues were considered as more informative than the facial features for signaling cooperative intents, and thus compensated lower levels of facial perceived trustworthiness. However, it is worth noting that if this has been the case, our data suggest that a switch from symbols- to facial features- based signaling of trustworthiness was not continuous throughout history, as our data highlight a non-monotonic increase of modeled perceived facial trustworthiness with time. On the other hand, the methodological choice of focusing on first impressions from faces is justified by the extensive work on social evaluations suggesting regularities in the way individuals perceive faces across cultures and in naturalistic contexts. Based on this bulk of evidence, it is probable that the way these cues are perceived have experienced less changes through time and cultures than culture-specific symbols such as the presence of a dog for communicating loyalty or parrots for symbolizing the Virgin Mary, paradise or only affluence and exotic places.

This limitation is the reason why, throughout the manuscript, we refer to our measure as “perceived *facial* trustworthiness” and not a more general ‘perceived trustworthiness’. Crucially, it seems a little bit confusing from Reviewer 2’s comment, whether our methods aimed at measuring trustworthiness or at approximating human ratings of perceived trustworthiness. We would thus like to take the opportunity to restate that, as noted in our manuscript, our goal is to approximate human evaluations of trustworthiness and we do not make any assumption or claim about the actual trustworthiness of the sitters whatsoever. Nonetheless, we agree with Reviewer 2 that our measure is modeled, and we added this notion using ‘modelled perceived facial trustworthiness’ in the revised version of the manuscript. We also included a discussion on our decision to focus on faces and its limits to the discussion section.

**4. General discussion**

Furthermore, the scores extracted by this method, while significant, are only moderately correlated with participants' ratings of trustworthiness. Future studies are needed to further identify the precise limitations of this method.

**4. General discussion**

Finally, it should be recognized that portraits contain much more information than just the facial features we analyzed in the present study. Pose, costume, the presence of certain objects or animals have been used throughout art history to signal certain statuses, characteristics, and intentions. For example, dogs are known to have been used in official portraiture as symbols of loyalty. Therefore, by relying solely on facial features, our analyses omitted culturally important information contained in portraits. One consequence of this methodological choice is that we cannot rule out the possibility that cooperative intentions were primarily communicated by means other than facial features in a more distant past and that their importance has gradually been replaced by that of facial features in more recent periods, a process that could explain the increase in perceived facial trustworthiness in more recent portraits. However, while the question of the robustness of first impressions of faces across populations and cultures requires further investigation, these other symbols are highly culture-specific (as in the case of parrots; Verdi, 2007), which precludes their use in longitudinal or comparative studies.

**Reference**

Verdi, Richard. *The parrot in art: from Dürer to Elizabeth Butterworth*. Scala Books, 2007.

*(3) the paper were framed narrowly as a replication attempt of the headline finding of Safra et al. (2020), without attempting to draw any conclusions about trustworthiness or speculating about why the findings did not replicate. This would mean removing the discussion of modernization theory from the introduction, the references to social trust in §2.3, and most especially the claims about the scientific utility of the method in the second paragraph of the general discussion.*

> We fully agree with reviewer 2 that this paper is a replication attempt of Safra et al. (2020). This is clearly indicated in the title and the abstract. In addition, more than half of the introduction is dedicated to highlighting the limitations of Safra et al. (2020) and how the present studies are more controlled in these aspects. We believe it is important to introduce the theoretical framework in which this study is integrated. More precisely, without this theoretical framework, the analyses of the facial features of the portraits and the correlations we make with economic variables are not only meaningless, but would give the false impression that science is about randomly fishing for patterns in the wild. In contrasts, our project is theory-driven, based on recent advances in psychological sciences, and its predictions have been preregistered.

Similarly, without discussing the scientific aim of this method, it is pointless to replicate Safra et al. (2020) or any other method, since the goal is not to develop new tools based on AI, but rather to show how advances in AI and the digital humanities in general can provide new insights into fundamental questions in psychological science. In the same vein, as with any replication attempt, it is crucial from an open science perspective to discuss why an initial finding replicates or does not replicate in order to better identify the limitations of the initial results, the methods, and what they reveal about human psychology.

To better account for these important methodological points, we justify the present replication and the use of the developed tool in the current scientific context, both in the introduction and in the discussion.

**1. General introduction**

Scientists must therefore rely on other sources of information to study psychological changes or regularities in the past and to test specific hypotheses about the sources of these changes. In economic history, scientists typically rely on administrative data, such as urbanization rates, literacy rates and average wages (A’Hearn et al., 2009; Bosker et al., 2013; Buringh & Van Zanden, 2009). These dataset provide valuable quantitative data about the dynamics of past societies, but they are limited in terms of insights into the psychological traits of individuals. On the other hand, historians have analyzed the content of cultural artifacts, be it codes of honor, common and canonical laws, books, portraits, or costumes (Duby, 1992; Elias, 1978). These works have provided important insights about the evolution of mentalities and values. However, they are mostly qualitative, which most often preclude the possibility to test hypotheses about the dynamics and the origins of the changes.

Thanks to recent advances in data sciences, it is now possible to move further (Atari & Henrich, 2022; Brand et al., 2019; Martins & Baumard, 2022; Morin & Acerbi, 2017). In this perspective, Baumard et al. (2022) relied on the analysis of book descriptions to study the evolution of the prevalence of love stories throughout time and space, showing causal relation between economic development and the rise of love stories. Focusing on the question of social trust, Martins & Baumard (2020) relied on the analysis of theatre play to study the evolution of moral norms in Europe between 1550 and the 1900. Similarly, in a recent article, Safra et al., (Safra et al., 2020) proposed to circumvent this difficulty by using another type of data: portraits. A portrait, just like a photograph or a selfie today, allows one to control the impression one wants to give to others.

**1. General introduction**

We also studied the underlying determinant of this increase in pro-sociality. In line with the modernization theory and previous work conducted on contemporary data (Inglehart, 2018; Inglehart & Welzel, 2005; Lipset, 1959), Safra et al. (2020) showed an association between economic resources and modeled perceived facial trustworthiness. We aimed at replicating this effect.

**4. General discussion**

Cognitive sciences and computational social sciences may have a lot to offer to cultural and economic history. They can use traditional material (i.e. portraits, but also texts, costumes, musics) in a new way to uncover cultural and potentially psychological traits that are crucial to understand the dynamics of past societies. Using insights from cognitive science studies in artworks are thus one more tool in the emerging field of quantitative psychological history (Acerbi et al., 2013; Baumard et al., 2022; Martins & Baumard, 2020, 2022; Seligman, 2022). Obviously, as with any scientific study, these findings must be taken with caution and require replication on different databases and, ideally, using different artifacts to assess their robustness. In the present study, we successfully replicated only one of the results reported by Safra et al. (2020) using an independent database in only one of our studies. As with any partial replication, this calls for a critical perspective on the limitations of the methods and the insights they can provide. In particular, it suggests that this methodology is still in its infancy and requires relatively large and well-balanced databases.

*5. The article is presented in an intelligible fashion and is written in standard English.*

*Yes.

6. The research meets all applicable standards for the ethics of experimentation and research integrity.
Not human subjects or animal research.*

*7. The article adheres to appropriate reporting guidelines and community standards for data availability.
- An OSF link is provided in the metadata, but currently it's set to private/only visible by permission. The authors should make this link public before the paper is accepted.
- For Study 1, the authors provide two OSF links in the text, to two frozen archives of the project. The archives include the monarch data scraped from Wikipedia, but do not include any portrait image data, intermediate non-copyrighted data (such as extracted facial action units or "perceived trustworthiness" scores), or analysis scripts. Study 1 does not appear to be computationally reproducible given the contents of this repository.
- For Study 2, the authors provide an OSF links in the text, a frozen archive of a distinct project. The project is also currently set to private/only visible by permission. The archive includes the portrait/subject metadata for the portraits in the sample, but again does not include portrait image data, intermediate data, or analysis scripts. Study 2 also does not appear to be computationally reproducible given the contents of this repository.*

We are sorry to learn that Reviewer 2 did not have access to the link provided in the rebuttal to Reviewer 1 first comments which provide a link to our data and scripts: <https://osf.io/buv7g/?view_only=6193f19fa053485b8db95bf8267c11f3> .

*7. PLOS authors have the option to publish the peer review history of their article (*[*what does this mean?*](https://journals.plos.org/plosone/s/editorial-and-peer-review-process#loc-peer-review-history)*). If published, this will include your full peer review and any attached files.*

*If you choose “no”, your identity will remain anonymous but your review may still be made public.*

***Do you want your identity to be public for this peer review?*** *For information about this choice, including consent withdrawal, please see our* [*Privacy Policy*](https://www.plos.org/privacy-policy)*.*

*Reviewer #1: No*

*Reviewer #2: No*
